# Candidaemia and cancer: patients are not all the same

**DOI:** 10.1186/1471-2334-6-50

**Published:** 2006-03-16

**Authors:** Alessandro Comarú Pasqualotto, Daniela Dornelles Rosa, Lidia Rosi Medeiros, Luiz Carlos Severo

**Affiliations:** 1 Medical School, The University of Manchester, UK; 2 Cancer Research UK, Department of Medical Oncology, Christie Hospital, Manchester, UK; 3 Post-Graduation in Epidemiology, Federal University of Rio Grande do Sul, Porto Alegre, Brazil; 4 Clinical Mycology Laboratory, Santa Casa Complexo Hospitalar, Porto Alegre, Brazil; 5 1/512 Wilmslow Road, M20 4BT, UK

## Abstract

**Background:**

Most of the studies about invasive *Candida *infections in cancer patients have focused on haematological patients. The aim of this study was to provide information about risk factors for candidaemia in patients with solid tumours.

**Methods:**

Retrospective cohort study. During a 9-year period (1995–2003) we reviewed all cases of candidaemia that affected cancer patients in Santa Casa Complexo Hospitalar, Brazil.

**Results:**

During the period of study, 210 patients had the diagnosis of candidaemia in our medical centre, and 83 of these patients had cancer (39.5%). The majority of patients with cancer had solid tumours (77.1%), mostly in the alimentary tract. Most of solid cancers were non-metastatic (71.9%). Major diagnoses in patients with haematological neoplasia were acute leukaemia (n = 13), high grade non-Hodgkin lymphoma (n = 5) and Hodgkin's disease (n = 1). Non-*Candida albicans *species caused 57.8% of the episodes of candidaemia in patients with cancer, mainly in patients with haematological malignancies (p = 0.034). Neutropenia and treatment with corticosteroids were more frequent in the haematological group, in comparison with patients with solid tumours. Only 22.2% of patients with solid tumours were neutropenic before candidaemia. Nonetheless, the presence of ileus and the use of anaerobicides were independent risk factors for candidaemia in patients with solid cancers. The overall mortality in cancer patients with candidaemia was 49.4%. We then compared 2 groups of adult patients with candidaemia. The first was composed of non-neutropenic patients with solid tumours, and the second group included patients without cancer. We found that central venous catheters and gastrointestinal surgery were independently associated with candidaemia in patients with solid tumour.

**Conclusion:**

Cancer patients with candidaemia seem to have very different predisposing factors to acquire the infection when stratified according to baseline diseases. This study provides some useful clinical information regarding risk for candidaemia in patients with solid tumours.

## Background

Most of the knowledge about invasive *Candida *infections in cancer patients has derived from patients with haematological malignancies [1], and data regarding risk factors in patients with solid tumours are sparse [2,3]. The purpose of this study was to compare the demographic features, risk factors, and aetiology of candidaemia amongst patients with solid tumours and those with haematological neoplasms in our medical centre over the last 9 years.

## Methods

A retrospective cohort study was performed in Santa Casa Complexo Hospitalar, a 1,200-bed Brazilian tertiary teaching hospital. During the period comprising 1995 to 2003, all patients with cancer who developed candidaemia were included in the study. Candidaemia was defined as the presence of at least one positive blood culture for *Candida *obtained from a peripheral vein in a patient with associated signs and symptoms temporally related [2]. The episode of candidaemia was considered outpatient-acquired if had occurred prior to or within 72 h of hospital admission [4]. Candidaemia in patients who had undergone major surgical procedures in the last 30 days or prosthesis insertion in the last year was considered to be nosocomial. Medical charts of these patients were reviewed to record clinical and demographic characteristics presented in the period of 30 days before the collection of the first positive blood sample for *Candida*. Patients were considered paediatrics if their age was ≤ 13 years-old [5]. Neutropenia was defined as an absolute neutrophils count < 1,000 cells/mm^3^. Breakthrough candidaemia was defined as the occurrence of candidaemia in a patient receiving at least 3 days of systemic antifungal therapy [6]. The protocol was approved by the hospital independent ethics committee (protocol 547/02). Due to the nature of this study there was no need to apply informed consent to patients.

Blood samples were processed with BacT/Alert™ automated system or lysis centrifugation (Isolator™). Only one isolate was included per patient (the first one). Germ tubes were performed, and negative strains were identified through kit ID 32C (BioMerieux, France).

### Statistical analysis

Descriptive statistics were used to summarize the data. Pearson's chi-square and Fisher's exact test were used to evaluate the association between qualitative variables, and Mann-Whitney test was used for the comparison of quantitative variables, with a bilateral level of significance of 5%. We used univariate analysis to identify candidate variables to include in the binary logistic regression model. A stepwise forward selection (Wald) logistic regression procedure was used to derive the model. We included only variables that showed a Wald test > 2 and a p-value < 0.25 in the univariate analysis [7]. Potential confounding variables were tested in multivariate analyses; if they changed the estimate of the association of interest, they remained in the final model. An important step in the process of modelling a set of data was determining whether or not there was evidence of interaction. Odds ratios with 95% confidence intervals were calculated. Data analysis was performed with the statistical software package SPSS version 10.0.

## Results

During the period of study, 210 patients had the diagnosis of candidaemia in our medical centre, and 83 of these patients had cancer (39.5%). The majority of these cancer patients were male (55.4%), and median age was 50.2 years old (range, 0.7–81.3 years old). Candidaemia in cancer patients was largely a nosocomial infection (89.2%).

Most of the patients with cancer had solid tumours (77.1%, n = 64). Most of the solid tumours affected the alimentary (36.0%, n = 23), genitourinary (20.4%, n = 13), and gynaecological tracts (18.8%, n = 12). Colon cancer was the most prevalent neoplasm (9.5%, n = 6). Uncommon cancers included tumours affecting the respiratory system (9.4%, n = 6) and sarcomas (4.8%, n = 3). Most of solid cancers were locally advanced (35.9%, n = 23), 31.3% were restricted to the affected organ (n = 20), and only 28.1% were metastatic (n = 18). Three patients (4.7%) had had complete response to treatment.

Major diagnoses in the group of patients with haematological neoplasia were acute leukaemia (68.4%, n = 13), high grade non-Hodgkin lymphoma (26.3%, n = 5) and Hodgkin's disease (5.3%, n = 1). Most of the patients with leukaemia were receiving induction or consolidation chemotherapy (61.6%, n = 8), 30.8% had progressive or resistant disease (n = 4), and 7.7% were in remission (n = 1). The patients with lymphoma were distributed in stages I or II disease (50.0%, n = 3) and stages III or IV (50.0%, n = 3).

The median number of positive blood cultures in cancer patients was 2 (range, 1–5) and median duration of candidaemia was 1.0 day (range, 1–20 days). Species other than *Candida albicans *were the aetiology of 57.8% of candidaemias in cancer patients, mainly *C. parapsilosis *(27.7%) and *C. tropicalis *(13.3%). *C. glabrata *and *C. krusei *were infrequent (3.6% each). Breakthrough candidaemia accounted for 9.6% of episodes (n = 8). Previous bacteraemia occurred in 32.5% (n = 27), mainly caused by gram-positive aerobic organisms (n = 16). *Candida *was isolated in sites other than blood in 34.9% of patients (n = 29), mainly from catheters (20.5%, n = 17), and urine (18.1%, n = 15). Concomitant bacteraemia occurred in 18.1% of cancer patients with candidaemia (n = 18). Central venous catheters (CVCs) were removed for 69.2% of cancer patients who had catheters at the moment of candidaemia, and median time for catheter removal was 5.0 days. Fundoscopic eye examination was performed for only 1 patient (1.2%). Systemic antifungal treatment was used in 75.9% of patients (n = 63), mostly amphotericin B desoxycholate (53.0%) or fluconazole (42.2%). The overall mortality was 49.4%.

Non-*Candida albicans *species caused 78.9% of episodes of candidaemia in patients with haematological malignancies and 51.6% in those with solid tumours (p = 0.034). The risk to have *C. albicans *as the aetiology of candidaemia for patients with solid tumours was 1.29 (CI 95% 1.03 to 1.61).

Table 1 shows the comparison of patients with haematological neoplasia and patients with solid tumours. At univariate analysis, haematological patients were more frequently exposed to corticosteroids and chemotherapy, in comparison to patients with solid tumours. The percentage of neutropenic patients was much higher in the haematological group – in fact, only 22.2% of patients with solid cancers had candidaemia following an episode of neutropenia. Age was also different between groups, with a higher proportion of children in the haematological group (47.4% versus 14.1%; p = 0.004). On the other side, patients with solid tumours had required major surgeries more often, mainly in the gastrointestinal tract. Median number of major surgical interventions for the solid tumour group was 2.0 (mean 1.6, range, 1–6), and median number of surgical procedures involving the gastrointestinal tract was 1.0 (range 1–5). Other risk factors more commonly found in the solid tumours group were ileus, use of anaerobicides, requirement of invasive mechanical ventilation, urinary catheters, and admission to the intensive care unit. Rates of antifungal therapy use and mortality were similar between these two groups. At multivariate analysis, the presence of ileus (OR 1.9; CI 95% 0.1 to 21.9) and use of anaerobicides (OR 1.2; CI 95% 0.2 to 6.5) were associated with a higher risk for candidaemia in patients with solid tumours. A significant positive interaction between these variables was found.

**Table 1:** Major demographic features, predisposing conditions, antifungal treatment and outcome for patients with candidaemia and solid tumours or haematological malignancies (univariate analysis).

Variables	Solid (%)	Haematological (%)	p value
Demographic factors			
Age (median, years)	54.2	14.7	0.007^c^
Male sex	51.6	68.4	0.194^a^
Nosocomial infection	89.1	89.5	1.000^b^
Predisposing conditions			
Neutropenia	22.2	78.9	< 0.001^a^
Duration of neutropenia (median, days)	5.0	17.0	0.004^c^
Mucositis	9.4	11.1	1.000^b^
Diarrhoea	29.7	21.1	0.460^a^
Ileus	34.4	5.6	0.016^a^
Corticosteroids	41.3	84.2	0.001^a^
Chemotherapy	29.4	84.2	< 0.001^a^
Previous stay in the intensive care unit	48.4	21.1	0.034^a^
Invasive medical procedures			
Central venous catheter	75.0	89.5	0.221^b^
Implanted port	25.0	36.8	0.311^a^
Mechanical ventilation	35.9	0.0	0.002^a^
Urinary catheter	59.4	10.5	< 0.001^a^
Parenteral nutrition	21.9	5.3	0.172^b^
Haemodialysis	4.7	5.3	1.000^b^
Radiotherapy	9.4	5.3	1.000^b^
Major surgery	42.2	0.0	0.001^a^
Gastrointestinal surgery	23.4	0.0	0.018^b^
Antibiotic use			
Duration (median, days)	13	15	0.996^c^
Number of antibiotics (median)	3	3	0.474^c^
Metronidazole or clindamycin	42.2	15.8	0.035^a^
4^th ^generation cephalosporins	29.7	68.4	0.003^a^
Glycopeptides	37.5	57.9	0.114^a^
Carbapenems	12.5	15.8	0.708^b^
Breakthrough candidaemia	7.8	15.6	0.376^b^
Previous bactaeremia	28.1	47.4	0.116^a^
Antifungal treatment	73.4	84.2	0.542^b^
Death during hospitalization	53.1	36.8	0.213^a^
Total (n)	64	19	83

^a ^Chi-square test; ^b ^Fisher's exact test; ^c ^Mann-Whitney test.

Based on these results, we were interested to document if patients with solid tumours had any particular risk factor for candidaemia in addition to the risk factors presented in other critically ill non-cancer patients. In order to do that, we decided to compare two groups: (1) non-neutropenic adult patients with solid tumours and candidaemia, and (2) all other adult patients with candidaemia seen in our medical centre during the same period, with diagnoses other than cancer. These groups were very similar. Patients without cancer (group 2) had a higher frequency of diagnoses such as chronic renal failure and chronic lung diseases. *Candida *was more commonly isolated from the urine in these patients. Severity of illness was comparable between groups, as judged by the APACHE II score and the proportion of patients with shock requiring inotropic support. At univariate analysis (table 2), the only variables more often found in group 1 were previous use of CVC – particularly implanted ports – and surgery involving the gastrointestinal tract. At multivariate analysis, again these two variables were associated with a higher risk for candidaemia in patients from group 1 (CVC: OR 1.9; CI 95% 0.8 to 6.4; gastrointestinal surgery: OR 3.1; CI 95% 0.1 to 57.0). The 95% confidence interval includes the null hypothesis, possibly because of the low number of patients in the groups of comparison. We found a significant positive interaction between these variables.

**Table 2:** Univariate analysis comparing non-neutropenic adult patients with candidaemia and solid tumours (group 1) and adult patients with candidaemia and other diagnoses than cancer (group 2).

Variables	Group 1 (%)	Group 2 (%)	p value
Demographic factors			
Age (median, years)	62.0	59.8	0.828^c^
Male sex	56.5	54.0	0.791^a^
Nosocomial infection	87.0	87.3	0.958^a^
Diabetes mellitus	15.2	28.6	0.101^a^
Chronic renal failure	6.5	20.6	0.040^a^
HIV infection	0.0	7.9	0.072^b^
Heart failure	2.2	11.1	0.135^b^
Liver disease	2.2	9.7	0.235^b^
Chronic obstructive pulmonary disease	4.3	25.4	0.003^a^
Predisposing conditions			
Transfusion of blood products	58.7	46.0	0.191^a^
Gastrointestinal bleeding	17.4	20.6	0.672^a^
Mucositis	6.5	0.0	0.072^b^
Diarrhoea	21.7	33.3	0.185^a^
Ileus	41.3	25.4	0.079^a^
Corticosteroids	28.3	46.0	0.060^a^
Previous bacteraemia	21.7	27.0	0.531^a^
Previous shock requiring vasopressors	23.9	31.7	0.371^a^
Previous stay in the intensive care unit	45.7	49.2	0.714^a^
Invasive medical procedures			
Central venous catheter	82.6	63.5	0.029^a^
Implanted port	21.7	0.0	<0.001^b^
Nephrostomy	6.5	1.6	0.308^b^
Mechanical ventilation	41.3	46.0	0.623^a^
Jejunostomy	10.9	6.3	0.489^b^
Urinary catheter	69.6	57.1	0.186^a^
Enteral feeding	54.3	54.0	0.969^a^
Chest drainage	13.0	11.1	0.759^a^
Abdominal drainage	23.9	12.7	0.127^a^
Parenteral nutrition	28.3	19.0	0.258^a^
Haemodialysis	6.5	15.9	0.137^a^
Major surgery	50.0	39.7	0.284^a^
Gastrointestinal surgery	32.6	14.3	0.023^a^
Neurosurgery	0.0	3.2	0.508^b^
Cardiothoracic surgery	6.5	9.5	0.731^b^
At candidaemia			
Shock requiring vasopressors	13.0	28.6	0.053^a^
APACHE II score (median)	15.0	16.5	0.742^c^
Concomitant bacteraemia	21.7	19.0	0.730^a^
Antibiotic use			
Duration (median, days)	11.5	12.0	0.513^c^
Number of antibiotics (median)	3.0	3.0	0.337^c^
Metronidazole or clindamycin	43.5	27.0	0.072^a^
4^th ^generation cephalosporins	34.8	31.7	0.739^a^
Glycopeptides	23.9	41.3	0.059^a^
Carbapenems	10.0	27.0	0.038^a^
*Candida *species			
*Candida albicans*	60.9	49.2	0.227^a^
*Candida parapsilosis*	17.4	22.2	0.535^a^
*Candida tropicalis*	10.9	12.7	0.771^a^
*Candida *isolation from sites other than blood			
Catheter	17.4	7.9	0.133^a^
Urinary	19.5	27.0	0.048^a^
Breakthrough candidaemia	6.5	4.8	0.696^b^
Antifungal treatment	71.7	71.4	0.972^a^
Death during hospitalization	58.7	55.6	0.744^a^
Total (n)	46	63	109

^a ^Chi-square test; ^b ^Fisher's exact test; ^c ^Mann-Whitney test.

## Discussion

Several studies have revealed risk factors for candidaemia amongst patients with cancer. Similar to other populations, the use of antibiotics is recognized as an important predisposing feature, as well as the presence of CVCs, neutropenia, surgery, parenteral nutrition, and corticosteroids [3,8-10]. However, as stated before, most of these risk factors were characterised in studies centred in patients with haematological malignancies. The main idea behind the present study was to provide some data for patients with solid tumours by comparing them with two other populations of patients with bloodstream *Candida *infection: patients with a malignant haematological disease and patients with diagnoses other than cancer. And, in fact, these populations appeared to be quite different regarding risk factors for candidaemia.

Invasive fungal infections are a major threat for patients with haematological malignancies. Furthermore, candidaemia is the most frequent bloodstream fungal infection affecting these individuals. Since neutrophils and mononuclear cells are very important to damage and kill yeast cells, hyphae and pseudohyphae, patients with haematological neoplasms are particularly at risk for these infections. This increased risk can either be a consequence of their underlying malignancy and/or the treatment for their disease [11], which frequently includes corticosteroids. According to previous studies [3,12], our haematological patients with candidaemia received chemotherapy more frequently than patients with solid tumours. In addition, previous use of corticosteroids was also more common in the haematological group. These data reinforce the idea that neutropenia and therapy with steroids are not crucial for the occurrence of candidaemia in patients with solid tumours. In a series of 20 patients with solid tumours and candidaemia treated with high-dose regimens of fluconazole [13], only one had previous chemotherapy. All patients had a CVC in place in that study. Although international guidelines [1] have applied the knowledge derived from patients with haematological malignancies to patients with solid tumours in the treatment of fever in patients with cancer, these populations are very heterogeneous, and specific risk factors need to be addressed to patients with solid tumours.

Different from other studies, which have found candidaemia more frequently in patients with haematological neoplasms [3,9,14], candidaemia in our institution occurred mainly in patients with solid tumours. This may have occurred because Santa Casa Complexo Hospitalar is a referral hospital for these conditions. Unfortunately, no denominator was available to calculate the incidence of candidaemia in different populations. The finding that patients with haematological malignancies were younger, in comparison with patients with solid tumours, may also just reflect the characteristics of patients treated in our medical centre.

Similarly to previous studies [3,8,12], our findings suggest that gastrointestinal surgery might be a predisposing factor to the development of candidaemia in patients with solid tumours. Firstly, ileus and use of anaerobicides were associated with a higher risk of candidaemia in patients with solid tumours, in comparison to haematological patients. Secondly, gastrointestinal surgery and the use of CVC were independently associated with risk for candidaemia in non-neutropenic adults with solid tumours, in comparison to other adult patients with candidaemia but no cancer. A significant positive interaction was observed for both comparisons, which means that patients who had ileus also received more anaerobicides, and those submitted to gastrointestinal surgery were also more frequently treated with CVC. This could merely be a reflection of the higher proportion of patients with gastrointestinal cancers in the solid tumour group, who were – as expected – treated surgically. However, it is also possible that the surgical trauma could have facilitated the translocation of these organisms to the bloodstream – in this case, gastrointestinal surgery is to be an independent risk factor for candidaemia in patients with solid tumours. New studies will be required to confirm this hypothesis, and to clarify if this is true only to patients with gastrointestinal cancers.

In accordance to the findings of Viscoli et al. [12], most of the patients with solid tumours and candidaemia in our study did not have metastatic disease, reinforcing the concept that candidaemia do not occur only in patients with advanced disease. The unexpected low frequency of fundoscopic examination amongst our cancer patients was discussed elsewhere [15]. The overall mortality amongst cancer patients with candidaemia was 49.4%, which was in accordance to other studies [16-21].

Likewise previous studies [3,9,22,23], species other than *Candida albicans *caused most episodes of candidaemia in cancer patients in our study, mainly in patients with haematological malignancies. The recognition of non-*Candida albicans *species as pathogens in oncology patients are supposed to be the result of the widespread use of antifungal agents in the early 1990's for prophylaxis after haematopoietic stem-cell transplantation and as the treatment in the setting of febrile neutropenia [24]. However, some studies [9,25] have reported low use of azoles in Brazil, suggesting that previous use of antifungals was not the main factor associated with the emergence of non-*Candida albicans *species in these institutions. In fact, many other factors seem to be implicated in the emergence of these species, which discussion is beyond the scope of this study. For instance, *C. parapsilosis *has been linked to the widespread use of CVCs [6,19], and *C. tropicalis *has been associated with cancer (especially neutropenia and mucositis) even before the introduction of fluconazole in clinical practice [9,26,27]. As previously showed [28], *Candida albicans *was the dominant species in surgical patients, as revealed for patients with solid tumours in this study.

This study has some limitations. It is important to emphasise that some of the differences here described might be related basically to differences in underlying diseases, as we did not have a control group without candidaemia. The lack of susceptibility tests is other drawback to be mentioned. The retrospective nature of this study might also have led to underestimation of important variables such as the grade of mucositis. It should also be noticed that the small number of patients included in this study might have prevented some associations to reach statistical significance. Additionally, this fact might also have influenced the interactions observed amongst variables in multivariate model. Although some risk factors for candidaemia in patients with solid tumours were here identified, there is a clear need for new studies in the field.

Our main conclusions are that cancer patients with candidaemia seem to have different predisposing factors to acquire the infection when stratified according to baseline diseases. Whilst neutropenia and therapy with corticosteroids were important risk factors for patients with haematological malignancies, multivariate analysis revealed that ileus and the use of anaerobicides were independently associated with candidaemia in patients with solid tumours. In the comparison to patients with candidaemia and diagnoses other than neoplasia, the performance of gastrointestinal surgery and the presence of CVC were found to be independent risk factors for candidaemia amongst non-neutropenic adult patients with solid cancers. When looking at the 95% confidence intervals, one might interpret the results as reflecting lack of association. However, that would be a mistaken conclusion because the likelihood of any given value of the true parameter being estimated is not uniform across the range of values contained in the confidence interval. The confidence interval merely expresses the statistical uncertainty of the point estimates. It is important to remember that statistical significance and the width of the confidence limits are strongly dependent on the sample size. In this study, there were few patients in some groups of comparison. In the same way, there was a significant interaction between the variables, meaning that the effects of the exposure on the outcomes differ depending on whether another variable (the effect modifier) is present. Interaction implies that the variables should be considered together, not separately. When interaction is present, the association between the risk factor and the outcome variable differs, or depends in some way on the level of the covariate, that is, the covariate modifies the effect of the risk factor [7].

Following a worldwide trend, species other than *C. albicans *were the main aetiology of candidaemia in this study. However, the majority of non-neutropenic adult patients with solid tumours had candidaemia caused by *C. albicans*. Although these results help to identify patients with solid tumours at higher risk for *Candida *bloodstream infections, large studies seem to be required to address specific risk factors for candidaemia in this population.

## Competing interests

The author(s) declare that they have no competing interests.

## Authors' contributions

ACP conceived the study, the design, co-ordinated it, reviewed the medical files, did the analyses, and prepared the final manuscript. DDR reviewed the medical files searching for oncological data, and worked in the analyses and preparation of the manuscript. LRM carried out the multivariate analysis and participated in the preparation of the manuscript. LCS supervised all the steps of the research. All authors saw the final manuscript and made contributions.

## Pre-publication history

The pre-publication history for this paper can be accessed here:


